# The Effectiveness of a Body-Affective Mindfulness Intervention for Multiple Sclerosis Patients with Depressive Symptoms: A Randomized Controlled Clinical Trial

**DOI:** 10.3389/fpsyg.2017.02083

**Published:** 2017-11-30

**Authors:** Sara Carletto, Valentina Tesio, Martina Borghi, Diana Francone, Francesco Scavelli, Gabriella Bertino, Simona Malucchi, Antonio Bertolotto, Francesco Oliva, Riccardo Torta, Luca Ostacoli

**Affiliations:** ^1^Department of Clinical and Biological Sciences, University of Turin, Turin, Italy; ^2^Department of Psychology, University of Turin, Turin, Italy; ^3^Neurologia 2 – Centro di Riferimento Regionale Sclerosi Multipla, Azienda Ospedaliero-Universitaria San Luigi Gonzaga, Orbassano, Italy; ^4^Clinical Psychology and Psychosomatics Service, Azienda Ospedaliero-Universitaria San Luigi Gonzaga, University of Turin, Orbassano, Italy; ^5^Clinical and Oncological Psychology, Città della Salute e della Scienza Hospital of Turin, Turin, Italy; ^6^Department of Neuroscience, University of Turin, Turin, Italy

**Keywords:** multiple sclerosis, depression, mindfulness, mindfulness based intervention, quality of life, psycho-education

## Abstract

**Purpose:** Mindfulness interventions have been shown to treat depressive symptoms and improve quality of life in patients with several chronic diseases, including multiple sclerosis, but to date most evaluation of the effectiveness of mindfulness interventions in multiple sclerosis have used patients receiving standard care as the control group. Hence we decided to evaluate the effectiveness of a group-based body-affective mindfulness intervention by comparing it with a psycho-educational intervention, by means of a randomized controlled clinical trial. The outcome variables (i.e., depression, anxiety, perceived stress, illness perception, fatigue and quality of life) were evaluated at the end of the interventions (T1) and after a further 6 months (T2).

**Methods:** Of 90 multiple sclerosis patients with depressive symptoms (Beck Depression Inventory-II score greater than 13) who were randomized, 71 completed the intervention (mindfulness group *n =* 36; psycho-educational group *n =* 35). The data were analyzed with GLM repeated-measures ANOVA followed by pairwise comparisons.

**Results:** Per-protocol analysis revealed a time by group interaction on Beck Depression Inventory-II score, with the mindfulness intervention producing a greater reduction in score than the psycho-educational intervention, both at T1 and at T2. Furthermore, the mindfulness intervention improved patients’ quality of life and illness perception at T1 relative to the baseline and these improvements were maintained at the follow-up assessment (T2). Lastly, both interventions were similarly effective in reducing anxiety and perceived stress; these reductions were maintained at T2. A whole-sample intention-to-treat (ITT) analysis broadly confirmed the effectiveness of the mindfulness intervention.

**Conclusion:** In conclusion, these results provide methodologically robust evidence that in multiple sclerosis patients with depressive symptoms mindfulness interventions improve symptoms of depression and anxiety and perceived stress, modulate illness representation and enhance quality of life and that the benefits are maintained for at least 6 months.

**Trial registration:** the study was registered in the ClinicalTrials.gov registry (NCT02611401).

## Introduction

Multiple sclerosis (MS) is a chronic, demyelinating disease and onset usually occurs at a young age. People with MS have to deal not only with many debilitating and unpredictable symptoms and with the loss of function and the consequent disability, but also with the unpredictability of the disease and the uncertain prognosis. This imposes a significant emotional burden and has a severe impact on psychosocial functioning.

Previous studies focusing on the psychosocial impact of MS have shown that depression, anxiety and reduced quality of life are prevalent (Wallin et al., 2006; Beiske et al., 2008; Byatt et al., 2011; Feinstein, 2011; Moore et al., 2012; Ostacoli et al., 2013). In particular, depression seems to be more prevalent in MS than in other chronic neurological diseases (Wallin et al., 2006) and three times more prevalent than in the general population (Feinstein, 2011). There are reports that depression affects from 15 to 47% of MS patients (Chwastiak et al., 2002; Siegert and Abernethy, 2005), with an estimated lifetime prevalence of 50% in people with MS (Sadovnick et al., 1991). The prevalence of comorbid depression in MS seems to worsen over time, although it is high even at the time of MS diagnosis (Marrie et al., 2015). The reasons for the comorbidity of MS and depression are many and complex. Depression could be considered a reaction to the unpredictability and chronicity of the disease, but it is also possible that MS-related biological processes such as immunological and inflammatory pathways, or psychosocial risk factors like inadequate coping or insufficient social support could predispose MS patients to depression (Feinstein, 2011; Feinstein et al., 2014; Boeschoten et al., 2017). Depression has a negative impact on the course of MS; as well as increasing the symptom burden and negatively influencing adherence to treatment it has direct pathophysiological effects on immunity (Mohr et al., 1997; Boeschoten et al., 2017). It has also been shown that depression in MS is strongly related to lower quality of life, cognitive dysfunction, elevated suicide risk and fatigue (Fruehwald et al., 2001; Brown et al., 2009; Patrick et al., 2009; Fiest et al., 2016; Boeschoten et al., 2017), which is one of the most commonly reported and debilitating symptoms of MS (Wallin et al., 2006). Nevertheless, affective disorders in MS are still under-recognized and under-treated by clinicians (Goldman Consensus Group, 2005). For these reasons the American Academy of Neurology formulated evidence-based recommendations for screening, diagnosis and treatment of psychiatric disorders in MS (Minden et al., 2014), recommending further research on the utility of treatments shown to be effective in other clinical populations.

Psychopharmacological treatments, such as serotonin selective reuptake inhibitors (SSRI), are quite effective as treatments for depression in MS, but they have prominent side effects and the drop-out rate is high (Koch et al., 2011). In contrast psychological treatments have been shown to have beneficial effects on both depression and quality of life in patients with MS. In particular, cognitive behavioral therapy (CBT) has been shown to have a moderate effect on depression (Thomas et al., 2006; Fiest et al., 2016). Feinstein and colleagues (Feinstein, 2011; Feinstein et al., 2014) have noted that publicly funded treatments for depression in MS patients must take account of resource constraints and that it is therefore crucial to identify brief, yet cost-effective interventions that can reduce depressive symptoms and the psychological burden of MS, in order to improve patients’ quality of life (Feinstein et al., 2014).

Mindfulness-based interventions (MBIs) are psychological treatments that meet the need for brevity and cost-effectiveness. They have been shown to be effective in patients with several diseases, including chronic pain, cancer and fibromyalgia (Hofmann et al., 2010; Crowe et al., 2016). MBIs influence emotion regulation, using awareness of the present moment and a non-judgmental, accepting attitude, to disrupting dysfunctional tendencies to avoid or over-engage with one’s disturbing physical sensations, emotions and thoughts. MBIs have been shown to have large beneficial effects on depression in patients with depressive disorders and to be effective in preventing relapse (Hofmann et al., 2010; Piet and Hougaard, 2011; Kuyken et al., 2016). Furthermore, MBIs have been shown to produce a moderate reduction in depressive symptoms associated with medical conditions (Hofmann et al., 2010; Crowe et al., 2016). These results suggest that MBIs could address processes underlying multiple disorders by changing several emotional and cognitive dimensions (Hofmann et al., 2010; Feinstein, 2011). Mindfulness can lead patients to relate to their physical and psychological symptoms in a different way, with a positive effect on coping strategies and adaptation to the disease (Feinstein, 2011).

Recent systematic reviews have indicated that MBIs are effective in MS patients (Simpson et al., 2014; Levin et al., 2014; San José et al., 2016). Both a review including only controlled trial (Simpson et al., 2014) and a review using a less restrictive criterion of effectiveness (Levin et al., 2014) concluded that MBIs can improve MS patients’ quality of life and mental health, and improve ability to cope with some of the physical symptoms, such as fatigue and pain. Senders et al. (2014) confirmed that in MS, trait mindfulness is related to lower psychological stress, a more constructive coping profile, increased resilience and higher quality of life. Bogosian et al. (2015) and Schirda et al. (2015) corroborated these results, suggesting that mindfulness could improve MS patients’ quality of life by reducing emotion dysregulation, especially in patients with more symptoms of depression. Moreover, MBIs were shown to have a low overall attrition rate and no side-effects and patients reported high goal satisfaction (Grossman et al., 2010; Simpson et al., 2014). However, systematic reviews have also highlighted that most of the data demonstrating the effectiveness of MBIs were obtained in non-controlled trials or in comparison with standard care and argued that there is a need for more rigorous clinical trials comparing MBI with active control groups (Levin et al., 2014; Simpson et al., 2014).

We therefore conducted a randomized controlled trial comparing a MBI with an active control intervention in MS patients with depressive symptoms. We evaluated the effects of a group-based body-affective mindfulness (BAM) intervention on depressive symptoms in patients with MS at the end of the intervention and 6 months later. We also evaluated the effects of the BAM intervention on quality of life, illness perception, symptoms of anxiety, perceived stress and fatigue. We hypothesized that the BAM intervention would be more effective than the control intervention, a psycho-educational intervention. The final aim of this study was to evaluate the effectiveness of the BAM intervention in treating depression and related symptoms and improving quality of life in caregivers. As the caregivers of patients with MS must deal with the stress and difficulties associated with management of a chronic disabling disease we expected that they would also benefit from the treatment.

## Materials and Methods

### Design

We carried out a randomized controlled clinical trial (RCT). MS patients with depressive symptoms were randomly allocated to the BAM group or the active control group, which received a psycho-educational intervention (PEI). The required sample size was calculated to be 82, based on α = 0.05, power = 0.80 and a medium effect size (0.25) with respect to depressive symptoms. The Medical Ethics Committee of San Luigi Gonzaga University Hospital approved the protocol and the study was registered in the ClinicalTrials.gov registry (NCT02611401). All the participants gave written, informed consent.

### Participants and Procedures

Participants were enrolled at the CReSM Unit of the San Luigi University Hospital of Orbassano (Italy), the regional referral center for MS.

Multiple sclerosis patients that presented to the CReSM for a routine visit, a blood test or an infusion therapy were invited to participate and complete the Beck Depression Inventory-II (BDI-II) on a consecutive basis. Patients with a BDI-II score greater than 13 who met the other criteria for participation were included in the study.

As previously described (Carletto et al., 2016), the inclusion criteria were: (1) definite diagnosis of MS (Mc Donald Criteria) (Polman et al., 2011) made or confirmed at the CReSM Unit at least 6 months prior the beginning of the study; (2) age between 18 and 65 years; (3) no evidence of clinical relapse and no worsening of score on the Expanded Disability Status Scale (EDSS) in the last 3 months; (4) an EDSS score lower than 6.5; (5) fluent Italian speaker; (6) legal capacity to consent to the treatment; (7) willingness to abstain from or to suspend all other psychological treatment; (8) suspension of all psychotropic medication at least 1 month before the start of the intervention or maintenance at baseline level throughout the study. The exclusion criteria were: (1) current serious psychological or psychiatric disorder, including severe major depressive disorder, psychotic disorder and bipolar disorder or active substance abuse as assessed by the Mini International Neuropsychiatric Interview-Plus (M.I.N.I.-Plus); (2) severe suicidality, including ideation, plan and intent; (3) presence of overt dementia; (4) corticosteroid treatment during the previous 30 days; (5) other serious medical disorders in addition to MS; (6) current pregnancy.

**FIGURE 1:**
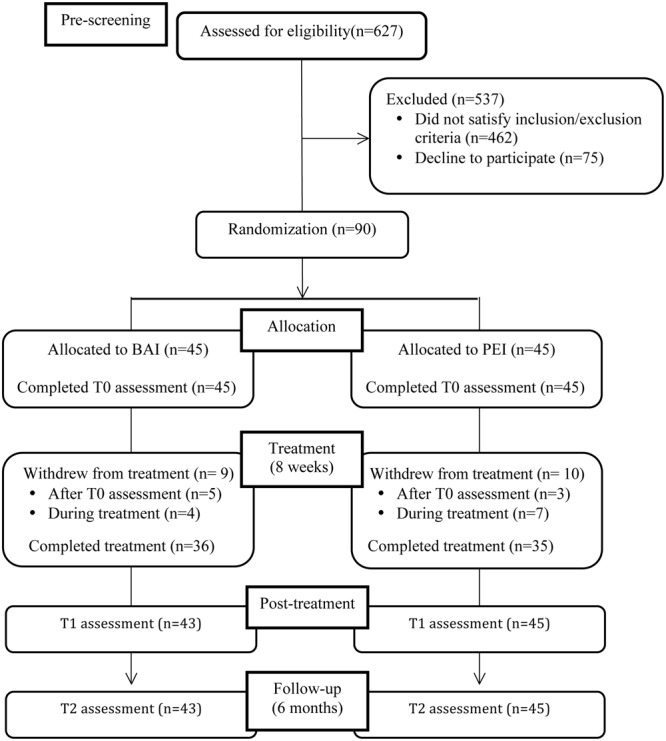
Participants flow diagram.

Of the 627 patients with MS who were pre-screened using the BDI-II, 462 did not satisfy the inclusion criteria and 75 refuse to participate (**Figure 1**). The final sample consisted of 90 patients. All patients were asked to invite a relative or caregiver to join their treatment group; 19 relatives agreed to participate in the treatment groups.

Patients and caregivers underwent three psychological assessments by trained clinical psychologists who were blind to group status: at baseline (T0), after the intervention (T1) and 6 months after the end of the intervention (follow-up, T2).

### Measures

The three psychological assessments included the administration of the following clinical interview and self-report questionnaires.

#### Fatigue Severity Scale (FSS)

This is a nine-item self-administered questionnaire, specifically developed to assess the severity of fatigue in various situations and to differentiate fatigue from clinical depression (Krupp et al., 1989). Scores greater than 36 suggest the presence of severe fatigue. The FSS has shown high Cronbach’s alpha values (0.84–0.95) in patients with MS (Rosti-Otajärvi et al., 2017).

#### Beck Depression Inventory-II (BDI-II)

This is a 21-item self-report instrument that assesses the presence and severity of depressive symptoms, based on the DSM-IV criteria (Beck et al., 1996). Total score ranges from 0 to 63, with higher scores indicating higher levels of depressive symptoms. The Consensus Group for Depression in MS (Goldman Consensus Group, 2005) and the American Academy of Neurology (Minden et al., 2014) consider the BDI-II (with a cut-off score of 13) the best method of screening for depression in MS patients. The internal consistency of the BDI-II is good to excellent (α = 0.83–0.96) (Wang and Gorenstein, 2013).

#### Beck Anxiety Inventory (BAI)

This is a 21-item self-report measure with high internal consistency (α = 0.92) that assesses the severity of cognitive, somatic and affective anxiety symptoms (Beck et al., 1988; Beck and Steer, 1993). Total score ranges from 0 to 63, with higher scores indicating higher levels of anxiety. Scores higher than 9 suggest the presence of clinical anxiety (10-16: mild anxiety; 17-29: moderate anxiety; ≥30: severe anxiety).

#### Perceived Stress Scale (PSS)

This is a 10-item questionnaire measuring the extent to which the respondent perceives his or her life situation as stressful (Cohen et al., 1983). Higher scores indicate a higher level of stress. The scale has shown an acceptable to excellent internal reliability (α = 0.78–0.91) (Cohen and Janicki-Deverts, 2012).

#### Brief Illness Perception Questionnaire (B-IPQ)

This is a nine-item self-report questionnaire that assesses emotional and cognitive representations of illness (Broadbent et al., 2006). Higher scores indicate a more negative perception of one’s illness. A recent systematic assessment of the reliability of the B-IPQ confirmed that it has good psychometric properties, including concurrent, predictive and discriminant validity (Broadbent et al., 2015).

#### Functional Assessment of Multiple Sclerosis (FAMS)

This is a self-report scale designed to assess six aspects of quality of life in patients with MS: Mobility, Symptoms, Emotional Wellbeing, General Contentment, Thinking and Fatigue, and Family/Social Wellbeing (Cella et al., 1996). Higher scores indicate better quality of life. The FAMS has good internal consistency, with Cronbach’s alpha for the subscales ranging from 0.82 to 0.96 (Smith and Driskell, 2016).

#### Mini International Neuropsychiatric Interview-Plus (M.I.N.I.-Plus)

This is a brief, structured diagnostic interview for the major Axis I psychiatric disorders compatible with the *Diagnostic and Statistical Manual of Mental Disorders*, 4th edition (DSM-IV) criteria (Sheehan et al., 1998). The M.I.N.I.-Plus has shown good inter-rater reliability and test–retest reliability (Sheehan et al., 1998).

Caregivers were asked to complete the BDI-II and the BAI to provide measures of their depressive and anxiety symptoms respectively. They also completed the PSS and the WHO-Quality of Life-Bref (WHOQOL-Bref; Murphy et al., 2000). The WHOQOL-Bref consists of 26 questions, two assess overall quality of life and the six relating to each of the following four domains: physical; psychological; social relationships; environment. The WHOQOL-Bref has shown good discriminant validity, test–retest reliability and internal consistency (α = 0.65-0.80) (De Girolamo et al., 2000).

### Randomization and Blinding

Patients were randomly assigned into the two intervention groups with a 1:1 ratio (45 in the BAI and 45 in the PEI group), using a block-wise randomization sequence (block size of 4 and 6). To ensure allocation concealment, the sequence was determined by an independent researcher blind to the initial assessment, using a random number generator^1^. To ensure the blinding of the clinical psychologists performing the assessments, the study coordinator communicated the treatment assignment to each patient.

Because family members or caregivers joined the group to which the corresponding patient had been randomly assigned it was not possible to stratify this variable; however we expected that the randomization of patients would ensure that participating caregivers were randomly and evenly distributed across groups.

### Intervention

The interventions were led by trained clinical psychologists who were blind to the results of the baseline assessment. Both interventions consisted of an 8-week group program, consisting of weekly 3-h sessions.

#### Body-Affective Mindfulness (BAM) Intervention

The MBI used in this study is called body-affective mindfulness (BAM); BAM is based on (1) awareness practices such as body scan, breath meditation, walking meditation and yoga exercises; (2) mindfulness in relationship practices such as loving kindness, enriching listening to nature and persons and self-compassion; (3) sensorimotor psychotherapy (Ogden et al., 2006). Sensorimotor psychotherapy emphasizes the use of somatic resources to attain and sustain a mindful disposition and integrates the concept of a stress response with the concept of a “window of tolerance” (Siegel, 2015), in order to maximize the clinical utility of the intervention and tailor it to MS patients (Keng et al., 2011). A more detailed description of the treatment protocol has been provided elsewhere (Carletto et al., 2016).

In addition to the eight weekly sessions, the BAM intervention also included an all-day (7-h) session and participants were required to carry out daily 45-min homework assignments, which consisted of mindfulness exercises and applications of mindfulness to everyday life.

#### Psycho-Educational Intervention (PEI)

We used a control intervention based on that used by Grossman et al. (2007) to control for the non-specific elements of the BAM treatment. The PEI was based on a psycho-educational framework and involved discussion of MS-related topics. The group practiced relaxation techniques and gentle stretching exercises at the end of each session. Participants were given handouts covering the topics discussed and encouraged to practice the exercises and techniques they had been shown as homework. The PEI was conducted by a psychotherapist with experience in relaxation training and in working with people with MS and followed the same weekly format as the BAM intervention, with the exception of the all-day session.

### Statistical Analysis

Data were analyzed using the Statistical Package for Social Science – Version 24 (SPSS-24; IBM SPSS Statistics for Macintosh, Version 24.0. Armonk, NY, United States: IBM Corp.).

Student’s *t*-test or the Mann–Whitney *U* test (where the Kolmogorov–Smirnov test indicated that distribution departed from normality) were used to analyze baseline group differences in continuous measures and Fisher’s Exact Test was used for categorical measures. The Mann–Whitney *U* test and Fisher’s Exact Test were also used for within-group baseline comparisons of the socio-demographic and clinical characteristics of completers and drop-outs.

GLM repeated-measures ANOVAs were conducted to evaluate the effects of group (BAM vs. PEI) and time (T0 vs. T1 vs. T2) and the time by group interaction, including only patients who completed the entire clinical trial according to the protocol [per-protocol (PP) analyses]. Mauchly’s test was used to assess violations of the sphericity assumption and the Greenhouse-Geisser correction was applied where necessary. Repeated-measures ANOVA is quite robust against violations of the assumptions of normality and sphericity (Stevens, 1986; Berkovits et al., 2000). When the sphericity violation is only slight (𝜀 > 0.75) applying the Greenhouse-Geisser correction provides a conservative assessment of differences (Berkovits et al., 2000).

Where significant effects were found, pairwise comparison were carried out applying the Bonferroni correction. Results of the *post hoc* comparison were presented as mean difference (MD) with 95% confidence interval (CI) and standard error (SE).

Finally, we performed an exploratory ITT analysis to take into account the missing data. All tests were two-sided and the level of significance was set at *p* < 0.05.

## Results

A flow diagram with the number of participants at each assessment stage is presented in **Figure 1**. Ninety patients were randomized: 45 were assigned to the BAM intervention and 45 to the PEI.

### Baseline Characteristics

There were no group differences in socio-demographic and clinical characteristics at baseline (**Tables 1, 2**). Patients reported moderate levels of depression and anxiety symptoms and high levels of fatigue and of perceived stress. Our sample also had generally negative illness representations and an unsatisfactory quality of life (**Table 2**).

**Table 1:** Demographic and clinical characteristics of randomized multiple sclerosis patients in the two intervention groups.

	BAM (*N* = 45)	PEI (*N* = 45)	Test	*p*
	Mean (SD)/Median (IQR)	Mean (SD)/Median (IQR)
Age	44.1 (9.4)	45.1 (9.3)	*t*(88) = -0.496	0.621^a^
Years of education	13 (2)	13 (5)	*U* = 899	0.340^b^
Time since diagnosis	9 (10)	7 (7)	*U* = 885	0.303^b^
EDSS	2 (1)	2.5 (2.3)	*U* = 806	0.127^b^

	**N (%)**	**N (%)**

Sex				1^c^
M	13 (28.9)	13 (28.9)
F	32 (71.1)	32 (71.1)
Marital status				0.739^c^
Single	15 (33.3)	12 (26.7)
Married	23 (51.1)	24 (53.3)
Divorced	7 (15.6)	9 (20)
Employment status				0.503^c^
Unemployed	13 (28.9)	17 (37.8)
Employed	31 (68.9)	28 (62.2)
Student	1 (2.2)	0 (0)
MS type				0.663^c^
Relapsing-remitting	38 (88.4)	36 (8%)
Primary progressive	1 (2.3)	1 (2.2)
Secondary progressive	3 (7)	4 (8.9)
Progressive relapsing	1 (2.3)	4 (8.9)
Pharmacological treatment	36 (80)	36 (80)		1^c^

*^a^*t*-test; ^b^Mann–Whitney *U* test; ^c^Fisher Exact test. MS, multiple sclerosis; EDSS, Expanded Disability Status Scale.*

**Table 2:** Comparison of clinical outcome measures at baseline between multiple sclerosis patients randomized in the two intervention groups.

	BAM (*N* = 45)	PEI (*N* = 45)		
	T0	T0	Test	*p*
FSS	50 (17)	51 (23)	*U* = 995	0.888^b^
Above cut-off	*35 (77.8%)*	*33 (73.3%)*	*X^2^*(1) = 0.241	0.807^c^
BDI-II	19 (11)	20 (10)	*U* = 887	0.310^b^
Above cut-off	*45 (100%)*	*45 (100%)*		
BAI	15 (17)	17 (18)	*U* = 919.5	0.453^b^
Above cut-off	*32 (71.1%)*	*33 (73.3%)*	*X^2^*(1) = 0.055	1^c^
PSS	25 (7)	24 (31)	*U* = 889	0.318^b^
B-IPQ	49.42 (8.83)	51.69 (9.72)	*t*(88) = -1.16	0.25^a^
FAMS				
*Total score*	109.6 (25.5)	104.2 (23.5)	*t*(87) = 1.02	0.309^a^
*Mobility*	20 (9)	16.5 (7)	*U* = 849	0.246^b^
*Symptoms*	19.33 (4.95)	19.81 (5.34)	*t*(87) = -0.434	0.665^a^
*Emotional wellbeing*	19.83 (7)	18 (6.75)	*U* = 818.5	0.158^b^
*General contentment*	14.44 (5.79)	13.48 (4.98)	*t*(87) = 0.845	0.401^a^
*Thinking and fatigue*	21 (10.5)	20.5 (6)	*U* = 914.5	0.535^b^
*Family/Social wellbeing*	17.73 (4.63)	16.22 (6.16)	*t*(79.8) = 1.3	0.196^a^
*Additional concerns*	33.95 (7.13)	32.46 (7.54)	*t*(87) = 0.956	0.342^a^

*^a^*t*-test; ^b^Mann–Whitney *U* test; ^c^Fisher Exact test. Mean (*SD*), Median (IQR), or *N*(%) are shown, accordingly. FSS, Fatigue Severity Scale; BDI-II, Beck Depression Inventory-II; BAI, Beck Anxiety Inventory; PSS, Perceived Stress Scale; B-IPQ, Illness Perception Questionnaire-Brief; FAMS, Functional Assessment of Multiple Sclerosis.*

After group allocation 5 of 45 patients (11.1%) assigned to the BAM and 3 of the 45 (6.7%) patients assigned to the PEI group declined to participate (**Figure 1**). Of the 82 patients who began the intervention, 4 (10%) (BAM) and 7 (16.7%) (PEI) failed to complete the intervention.

A comparison of completers and drop-outs found no baseline difference in the socio-demographical and clinical characteristics of those assigned to BAM intervention. In the case of the PEI group there were baseline differences between completers and drop-outs with respect to BAI score (*U* = 82.5, *p* = 0.011), PSS score (*U* = 89.5, *p* = 0.019) and Thinking and Fatigue subscale of the FAMS score (*U* = 91.5, *p* = 0.027), with drop-outs having lower anxiety [Mdn (IQR) = 9 (13) vs. 19.5 (19)] and lower perceived stress [Mdn = 14 (13) vs. 25 (10)] than completers as well as better quality of life in terms of the Thinking and Fatigue subscale [Mdn (IQR) = 23.5 (8) vs. 19 (5.25)].

### Per-protocol Analyses

We compared the impact of the interventions on the clinical outcome variables in completers (BAM: *N* = 36; PEI: *N* = 35) (**Tables 3, 4**).

**Table 3:** Repeated measures ANOVA on multiple sclerosis patients’ clinical outcome measures (per-protocol analyses).

		T0	T1	T2	F	*p*	$\eta_{p}^{2}$
FSS	BAM	46.25 (11.55)	40.92 (13.46)	41.61 (13.46)	Time: *F*(2,138) = 2.21	0.113	0.031
	PEI	45.23 (15.46)	45.51 (12.21)	45.69 (14.53)	Group: *F*(1,69) = 0.81	0.373	0.012
					T × G: *F*(2,138) = 7.93	0.057	0.041
BDI-II	BAM	21.39 (7.38)	10.28 (9.05)	10.03 (7.42)	Time: *F*(2,138) = 64.43	<0.001	0.487
	PEI	22.63 (7.36)	17.51 (11.08)	16.11 (11.32)	Group: *F*(1,69) = 6.45	0.013	0.086
					T × G: *F*(2,138) = 6.79	0.002	0.090
BAI	BAM	17.06 (11.09)	14.19 (11.84)	16.17 (10.18)	Time: *F*(2,138) = 3.46	0.034	0.048
	PEI	20.8 (11.43)	17.71 (10.94)	18.11 (10.84)	Group: *F*(1,69) = 1.82	0.181	0.026
					T × G: *F*(2,138) = 0.37	0.692	0.005
PSS	BAM	23.92 (6.83)	18.33 (7.68)	20.19 (7.3)	Time: *F*(2,138) = 10.79	<0.001	0.135
	PEI	24.2 (7.28)	22.37 (5.89)	22.94 (6.94)	Group: *F*(1,69) = 2.94	0.091	0.041
					T × G: *F*(2,138) = 2.75	0.067	0.038
B-IPQ	BAM	49.81 (9.04)	44.92 (9.48)	44.94 (9.59)	Time: *F*(1.77,122.2) = 7.54	0.001	0.098
	PEI	51.86 (10.34)	50.54 (10.51)	51.54 (10.02)	Group: *F*(1,69) = 5.06	0.028	0.068
					T × G: *F*(1.77,122.17) = 30.91	0.027	0.054

*T × G, time by group interaction effect. FSS, Fatigue Severity Scale; BDI-II, Beck Depression Inventory-II; BAI, Beck Anxiety Inventory; PSS, Perceived Stress Scale; B-IPQ, Illness Perception Questionnaire-Brief.*

**Table 4:** Repeated measures ANOVA on multiple sclerosis patients’ quality of life assessed with the Functional Assessment of Multiple Sclerosis questionnaire (per-protocol analyses).

		T0	T1	T2	F	*p*	$\eta_{p}^{2}$
*Total score*	BAM	110.9 (23.61)	121 (25.76)	120.4 (24.17)	Time: *F*(2,136) = 3.25	0.042	0.046
	PEI	101.3 (24.42)	101.5 (25.55)	97.35 (25.04)	Group: *F*(1,68) = 10.32	0.002	0.132
					T × G: *F*(2,136) = 7.63	0.001	0.101
*Mobility*	BAM	18.31 (5.64)	19.19 (5.53)	18.86 (5.67)	Time: *F*(2,136) = 0.55	0.579	0.008
	PEI	16.28 (5.38)	15.92 (5.26)	15.16 (5.21)	Group: *F*(1,68) = 6.5	0.013	0.087
					T × G: *F*(2,136) = 2.12	0.125	0.030
*Symptoms*	BAM	19.33 (4.34)	20.33 (4.43)	20.5 (4.13)	Time: *F*(2,136) = 0.19	0.831	0.003
	PEI	19.57 (5.33)	18.27 (5.21)	18.35 (5.86)	Group: *F*(1,68) = 1.67	0.200	0.024
					T × G: *F*(2,136) = 5.5	0.005	0.075
*Emotional wellbeing*	BAM	19.69 (5.38)	22.31 (5.24)	21.94 (5.69)	Time: *F*(1.76,120.1) = 7.68	0.001	0.101
	PEI	17.09 (5.41)	18.57 (5.58)	17.71 (5.91)	Group: *F*(1,68) = 9.3	0.003	0.120
					T × G:F(1.76,120.1) = 1.44	0.241	0.021
*General contentment*	BAM	14.86 (6.07)	16.78 (5.75)	17.11 (5.67)	Time: *F*(1.74,118.4) = 3.54	0.038	0.049
	PEI	13.09 (5.05)	13.63 (5.33)	12.71 (4.77)	Group: *F*(1,68) = 6.88	0.011	0.092
					T × G: *F*(1.74,118.38) = 3.93	0.027	0.055
*Thinking and fatigue*	BAM	20.81 (7.31)	24.03 (7.16)	23.78 (5.81)	Time: *F*(2,136) = 2.60	0.078	0.037
	PEI	19.33 (6.80)	19.11 (7.91)	18.43 (7.88)	Group: *F*(1,68) = 6.72	0.012	0.090
					T × G: *F*(2,136) = 6.51	0.020	0.087
*Family/social wellbeing*	BAM	17.89 (4.48)	18.39 (5.35)	18.22 (4.51)	Time: *F*(2,136) = 0.87	0.423	0.013
	PEI	15.97 (6.47)	16.01 (6.11)	15 (6.18)	Group: *F*(1,68) = 4.17	0.050	0.058
					T × G: *F*(2,136) = 1.13	0.326	0.016
*Additional concerns*	BAM	33.91 (7.37)	35.36 (8.53)	34.81 (7.93)	Time: *F*(2,136) = 0.36	0.700	0.005
	PEI	32.31 (8.16)	31.66 (8.74)	32.6 (8.45)	Group: *F*(1,68) = 2.25	0.138	0.032
					T × G: *F*(2,136) = 1.67	0.191	0.024

*T × G, time by group interaction effect.*

#### Depressive Symptoms (**Table 3**)

There were group and time effects on BDI-II as well as a medium-sized group by time interaction (*p* < 0.002; $\eta_{p}^{2}$ = 0.09). Compared with baseline, both interventions reduced depressive symptoms at T1 (BAM: MD = 11.11, 95% CI: 8.17 – 14.57, *SE* = 1.2, *p* < 0.001; PEI: MD = 5.11, 95% CI: 2.13 – 8.1, *SE* = 1.2, *p* < 0.001). In both groups the improvement remained stable at the follow-up (BAM: MD = 0.25, 95% CI: -2.49 – 2.99, *SE* = 1.12, *p* = 1; PEI: MD = 1.4, 95% CI -1.38 – 4.18, *SE* = 1.13, *p* = 0.66). However, pairwise comparison revealed that BAM intervention was more effective than the PEI, at T1 (MD = -7.24, 95% CI: -12.02 – -2.45, *SE* = 2.4, *p* = 0.004) and at T2 (MD = -6.09, 95% CI: -10.61 – -1.57, *SE* = 2.27, *p* = 0.009).

#### Fatigue (**Table 3**)

There was no effect of time or group on FSS score, indicated that neither intervention improved fatigue symptoms.

#### Anxiety Symptoms (**Table 3**)

There was a medium-sized main effect of time on BAI score. Pairwise comparison confirmed an overall reduction in symptoms between baseline and T1 (MD = 2.97, 95% CI: 0.120 – 5.83, *SE* = 1.16, *p* = 0.038), which remained stable between T1 and T2 (MD = -1.19, 95% CI: -3.78 – 1.41, *SE* = 1.06, *p* = 0.796). However, the BAM intervention was no more effective than the PEI, i.e., there was no time by group interaction.

#### Perceived Stress Symptoms (**Table 3**)

There was a medium-sized main effect of time on PSS score, such that both interventions reduced perceived stress at T1 relative to baseline (MD = 3.71, 95% CI: 1.72 – 5.69, *SE* = 0.81, *p* < 0.001). The reduction remained stable between T1 and T2 (MD = -1.22, 95% CI: -3.15 – 0.72, *SE* = 0.79, *p* = 0.380). There was no main effect of group and no time by group interaction.

#### Illness Perception (**Table 3**)

There were medium-sized main effects of time and group on B-IPQ score. There was a time by group interaction, reflecting the lack of change in perception of illness in the PEI group (T0 *vs.* T1: MD = 1.31, 95% CI: -1.71 – 4.34, *SE* = 1.23, *p* = 0.869; T1 *vs.* T2: MD = -1, 95% CI -3.47 – 1.47, *SE* = 1, *p* = 0.969). In contrast the BAM intervention improved B-IPQ score at T1 (MD = 4.89, 95% CI: 1.91 – 7.87, *SE* = 1.21, *p* < 0.001) and this improvement that remain stable at T2 (MD = -0.03, 95% CI -2.49 – 2.4, *SE* = 0.99, *p* = 1); in other words patients rated their illness as less threatening after the BAM intervention than at baseline.

#### Quality of Life (**Table 4**)

Repeated-measures ANOVA showed a medium-sized time by group interaction size with respect to the total score of the FAMS. The BAM intervention, but not the PEI, improved patients’ quality of life at T1 relative to baseline (MD = -10.14, 95% CI: -16.19 – -4.09, *SE* = 2.47, *p* < 0.001) and this improvement remained stable at T2 (MD = 0.62, 95% CI: -5.37 – 6.61, *SE* = 2.44, *p* = 1).

There were time by group interactions with respect to scores on the Symptoms, General Contentment, and Thinking and Fatigue subscales, these effects ranged from medium to small in size. Pairwise comparisons confirmed that the BAM intervention, but not the PEI, led to improvements at T1 relative to baseline (General Contentment: MD = -1.92, 95% CI: -3.39 – -0.44, *SE* = 0.6, *p* = 0.006; Thinking and Fatigue: MD = -3.22, 95% CI: -5.48 – -0.97, *SE* = 0.92, *p* = 0.002), that were maintained at T2 (General Contentment: MD = -0.33, 95% CI: -1.75 – 1.09, *SE* = 0.58, *p* = 1; Thinking and Fatigue: MD = 0.25, 95% CI: -1.73 – 2.23, *SE* = 0.81, *p* = 1). In the case of the Symptoms subscale pairwise comparisons revealed no differences.

There was a main effect of group on Mobility subscale score, with higher scores overall in the BAM group than the PEI group (MD = 3.06, 95% CI: -3.39 – -0.44, *SE* = 1.2, *p* = 0.013). Finally, there were medium-sized main effects of time and group on Emotional Wellbeing subscale scores. Pairwise comparisons showed that overall emotional wellbeing was better in the BAM group than the PEI group (MD = 3.6, 95% CI: 1.24 – 5.95, *SE* = 1.18, *p* = 0.003). There were also overall increases in scores between baseline and T1 (MD = -1.94, 95% CI: -3.09 – -0.8, *SE* = 0.47, *p* < 0.001), suggesting that both groups showed improvements in emotional wellbeing.

#### Psychopathology

At baseline the results of the M.I.N.I.-Plus suggested that adjustment disorders (BAM: 47.2%; PEI: 28.6%), anxiety disorders (including generalized anxiety disorders, panic disorders, phobia, etc.) (BAM: 33.3%; PEI: 20%) and major depressive episode (BAM: 30.6%; PEI: 37.1%) were prevalent in both groups. The proportion of participants no longer meeting the criteria for these disorders after the intervention was higher in the BAM group than the PEI group (see Supplementary Table 1).

### ITT Analyses (**Table 5**)

**Table 5:** Repeated measures ANOVA on the entire randomized multiple sclerosis patients (ITT analyses).

		T0	T1	T2	*F*	*p*	$\eta_{p}^{2}$
FSS	BAM	46.62 (10.91)	41.53 (12.79)	42.2 (12.75)	Time: *F*(2,176) = 4.6	0.011	0.050
	PEI	45.38 (14.93)	44.36 (13.02)	44.84 (14.83)	Group: *F*(1,88) = 0.31	0.577	0.004
					T × G: *F*(2,176) = 2.3	0.103	0.026
BDI-II	BAM	20.91 (7.41)	11.71 (9.84)	11.47 (9.50)	Time: *F*(1.81,158.9) = 65.6	<0.001	0.427
	PEI	21.64 (7.06)	16.22 (10.45)	14.6 (10.79)	Group: *F*(1,88) = 2.6	0.111	0.029
					T × G: *F*(1.81,158.9) = 2.94	0.061	0.032
BAI	BAM	16.71 (10.93)	14.31 (12.13)	15.31 (10.19)	Time: *F*(2,176) = 2.15	0.119	0.024
	PEI	18.56 (11.34)	17.16 (10.94)	16.67 (10.27)	Group: *F*(1,88) = 1	0.319	0.011
					T × G: *F*(2,176) = 0.29	0.747	0.003
PSS	BAM	23.89 (6.8)	18.4 (7.77)	19.71 (7.52)	Time: *F*(2,176) = 11	<0.001	0.111
	PEI	22.69 (7.64)	21.4 (6.39)	21.89 (7.03)	Group: *F*(1,88) = 1.12	0.292	0.013
					T × G: *F*(2,176) = 4.43	0.013	0.048
B-IPQ	BAM	49.42 (8.83)	45.58 (9.45)	44.96 (9.31)	Time: *F*(1.73,152.2) = 8.95	<0.001	0.092
	PEI	51.69 (9.72)	49.71 (10.05)	50.82 (9.40)	Group: *F*(1,88) = 5.21	0.025	0.056
					T × G: *F*(1.73,152.2) = 2.78	0.073	0.031
FAMS							
*Total score*	BAM	109.6 (25.5)	119.3 (28.30)	119.1 (27.1)	Time: *F*(2,174) = 5.54	0.005	0.060
	PEI	104.2 (23.50)	105.7 (24.80)	103.3 (26.4)	Group: *F*(1,87) = 5.24	0.025	0.057
					T × G: *F*(2,174) = 5.76	0.004	0.062
*Mobility*	BAM	17.87 (6.01)	18.67 (5.84)	18.16 (6.09)	Time: *F*(2,174) = 0.52	0.593	0.006
	PEI	17.01 (5.50)	16.34 (5.26)	16.07 (5.6)	Group: *F*(1,87) = 2.51	0.117	0.028
					T × G: *F*(2,174) = 2.59	0.078	0.029
*Symptoms*	BAM	19.33 (4.95)	20.29 (5.04)	20.87 (4.62)	Time: *F*(2,174) = 0.33	0.722	0.004
	PEI	19.81 (5.34)	18.94 (5.31)	18.77 (5.82)	Group: *F*(1,87) = 1.02	0.315	0.012
					T × G: *F*(2,174) = 6.71	0.002	0.072
*Emotional wellbeing*	BAM	19.13 (5.38)	21.65 (5.72)	21.53 (5.88)	Time: *F*(1.75,151.9) = 11.21	<0.001	0.114
	PEI	17.36 (5.54)	19.2 (5.43)	18.3 (5.62)	Group: *F*(1,87) = 5.78	0.018	0.062
					T × G: *F*(1.75,151.9) = 1.22	0.294	0.014
*General contentment*	BAM	14.44 (5.79)	16.36 (5.87)	16.62 (5.98)	Time: *F*(1.67,145.4) = 6.02	0.005	0.065
	PEI	13.48 (4.98)	14.24 (5.34)	13.95 (5.33)	Group: *F*(1,87) = 3.27	0.074	0.036
					T × G: *F*(1.67,145.4) = 1.95	0.153	0.022
*Thinking and fatigue*	BAM	21.07 (7.63)	24.04 (7.93)	23.93 (6.6)	Time: *F*(2,174) = 4.39	0.014	0.048
	PEI	20.37 (6.77)	20.16 (7.92)	20.33 (8.32)	Group: *F*(1,87) = 3.44	0.067	0.038
					T × G: *F*(2,174) = 5.21	0.006	0.057
*Family/Social Well-Being*	BAM	17.73 (4.63)	18.24 (5.10)	17.96 (4.88)	Time: *F*(2,174) = 0.89	0.413	0.010
	PEI	16.22 (6.16)	16.54 (5.92)	15.84 (5.98)	Group: *F*(1,87) = 2.77	0.100	0.031
					T × G: *F*(2,174) = 0.31	0.737	0.004
*Additional Concerns*	BAM	33.95 (7.13)	35.04 (8.63)	35.07 (7.94)	Time: *F*(1.87,162.9) = 1.06	0.346	0.012
	PEI	32.46 (7.54)	32.62 (8.15)	33.21 (7.8)	Group: *F*(1,87) = 1.85	0.178	0.021
					T × G: *F*(1.87,162.9) = 0.50	0.597	0.006

*T × G, time by group interaction effect; FSS, Fatigue Severity Scale; BDI-II, Beck Depression Inventory-II; BAI, Beck Anxiety Inventory; PSS, Perceived Stress Scale; B-IPQ, Illness Perception Questionnaire-Brief; FAMS, Functional Assessment of Multiple Sclerosis.*

An ITT analysis was also performed on the whole randomized sample. Only two patients (they were amongst those who declined to participate in the BAM group) refused to complete the T1 and T2 assessments. In the case of these two patients baseline data were substituted for the missing T1 and T2 data. The results generally confirmed the PP findings on the effectiveness of the BAM, especially with regard to quality of life (FAMS). In the ITT analyses the time by group interaction for BDI-II score was no longer significant (*p* = 0.061), although in descriptive terms it was only the BAM group which had a mean score below the threshold after the intervention. The ITT analyses did show, however, a main effect of time on FSS score [*F*(2,176) = 4.6, *p* = 0.011; $\eta_{p}^{2}$ = 0.050], reflecting an overall reduction in fatigue between baseline and T1 (MD = 3.06, 95% CI: 0.386 – 5.73, *SE* = 1.1, *p* = 0.019). In the ITT analysis of illness perception there was no longer a time by group interaction. There was, however, a time by group interaction with respect to PSS score reflecting a reduction in perceived stress symptoms relative to baseline in the BAM group both at T1 (MD = 5.49, 95% CI: 2.91 – 8.07, *SE* = 1.06, *p* < 0.001) and at T2 (MD = 4.18, 95% CI: 1.44 – 6.92, SE = 1.12, *p* = 0.001).

### Caregivers

Nineteen caregivers agreed to participate in the same intervention group as the patient for whom they cared (BAM *n* = 9; PEI: *n* = 10). Half the participating caregivers (50%) were the mothers of MS patients (see Supplementary Table 2 for further details). One caregiver dropped out after the baseline assessment and another dropped out during the intervention phase. Due to the low number of caregivers that agreed to participate we were unable to evaluate the effects of the interventions on this population.

## Discussion

To the best of our knowledge, this is the first RCT to evaluate the effectiveness of a group-based MBI in MS patients with depressive symptoms by comparing it with a control intervention. In MS depression is one of the main determinants of patients’ quality of life. It can increase fatigue and further compromise cognitive function and may also have a negative impact on relationships and reduce adherence to medication (Feinstein, 2011). For these reasons the Goldman Consensus Conference (Goldman Consensus Group, 2005) and the American Academy of Neurology (Minden et al., 2014) have highlighted the importance of diagnosing and treating depression in people with MS.

Mindfulness-based interventions are a relatively brief and cost-effective psychological treatment that have been shown to reduce depressive symptoms and improve quality of life in patients with several chronic diseases (Hofmann et al., 2010; Crowe et al., 2016; Veehof et al., 2016; Haller et al., 2017). Recent systematic reviews concluded that MBIs could also help patients with MS, by reducing the mental (such as anxiety and depression) and physical (i.e., fatigue) symptoms and improving the patients’ quality of life (Simpson et al., 2014; Levin et al., 2014; San José et al., 2016). However, none of these studies compared MBIs with an active control, which means that there are question marks over the specificity of the efficacy of MBIs.

When only patients who completed the interventions are considered, our RCT data indicate that the BAM intervention was more effective in reducing symptoms of depression (as measured by the BDI-II) than the PEI. Although both groups showed improvements in BDI-II score after the intervention and at the follow-up assessment, the BAM had lower BDI-II scores; this was a medium-sized effect. This finding extends previous research showing that in MS patients MBIs are effective when compared with standard care (Mills and Allen, 2000; Grossman et al., 2010; Tavee et al., 2011), by showing that a MBI was more effective than a psycho-educational intervention in reducing symptoms of depression. In the ITT analysis only the main effect of time remained statistically significant, weakening the evidence for the superior efficacy of the BAM intervention relative to the PEI. However, given that depressive symptoms can remit or reduce spontaneously (Posternak and Miller, 2001), it is possible that this occurred in the patients who dropped out, reducing the difference between the two interventions and masking the superior efficacy of the BAM that was found in the PP analysis. It should be noted that in both the PP and ITT analyses, the BAM group’s mean BDI-II score was below the threshold after the intervention, indicating the BAM intervention had a clinically meaningful impact on depressive symptoms.

The BAM-induced reduction in depressive symptoms as assessed by the BDI-II, which is a self-report measure, was replicated in the results from the semi-structured clinical interview (M.I.N.I.-Plus). Previous studies have relied on self-report outcome measures (Levin et al., 2014; Simpson et al., 2014) and no previous studies have evaluated psychosocial functioning before and after an intervention using objective measures. The M.I.N.I.-Plus data confirmed that in the BAM group there was a reduction in the number of patients currently experiencing a major depressive episode or adjustment disorders after the intervention. The M.I.N.I.-Plus also showed a reduction in the number of patients in the BAM group who met the criteria for an anxiety disorder (generalized anxiety disorder, panic disorders or specific phobia). At the baseline assessment both groups reported mild to moderate levels of anxiety, in addition to high perceived stress.

The BAM intervention was no more effective than the PEI in reducing anxiety symptoms and perceived stress, according to the self-report data (BAI and PSS). Both treatments reduced symptoms and the effects were maintained at the follow-up assessment, suggesting that they were similarly effective in producing long-term improvements in these domains.

A recent review indicated that relative to no treatment or control treatments such as standard care or walking in the garden, MBIs have a positive impact on fatigue in neurological conditions such as stroke, traumatic brain injury and MS (Ulrichsen et al., 2016). In our study the BAM and PEI interventions were not effective (PP analysis) or only similarly effective (ITT analysis) in reducing fatigue. Further research into this area is needed.

After receiving a diagnosis of illness, individuals develop an organized pattern of cognitive and emotional representations of their own illness, which can be referred to as an illness perception (Petrie and Weinman, 2006). These beliefs can influence coping behavior (Leventhal et al., 1984), with negative illness perceptions being associated with poorer recovery and increased healthcare use, even after controlling for objective illness severity (Leventhal et al., 1984; Petrie and Weinman, 2006). Our PEI was not effective in modifying MS patients’ illness perception, whereas patients in the BAM group reported less negative illness perceptions after the intervention, suggesting that the intervention had promoted a positive reorganization of their cognitive and emotional illness representations. A change in illness perceptions can help to reduce everyday disability and improve functioning and quality of life (Petrie and Weinman, 2006).

Mindfulness-based interventions have been shown to enhance quality of life in many different chronic conditions (Demarzo et al., 2015; Crowe et al., 2016; Veehof et al., 2016; Haller et al., 2017) and we confirmed this with respect to MS in our study. We found that the BAM intervention was effective in improving patients’ overall quality of life, whereas the PEI was not. Furthermore, the improvement seen in the BAM group was maintained 6 months after the intervention had ended, suggesting that the BAM intervention induces long-term modifications in quality of life. In particular, the BAM intervention reduced problems with concentration, thinking and fatigue and improved scores on the general contentment subscale, which deals with satisfaction and acceptance of health-related quality of life. The only quality of life domain to show improvement in the PEI group was emotional wellbeing, and the BAM group showed a similar improvement.

Mindfulness-based interventions have been shown to have a low attrition rate and no side effects (Grossman et al., 2010; Simpson et al., 2014). In our study the two interventions had comparable attrition rates. Eight patients dropped out after group allocation (5 from the BAM group; 3 from the PEI group) and 11 patients dropped out during the treatment (3 from the BAM group; 7 from the PEI group). The majority of the latter category dropped out due for logistic or work-related reasons. Although the attrition rate was close to the acceptable threshold, which is 20% of randomized participants (Schulz and Grimes, 2002), the only baseline differences between completers and dropouts, were lower levels of anxiety and perceived stress in patients that dropped out of the PEI. Furthermore, only 4% of patients allocated to the BAM group refused to complete the post-treatment assessments, which allowed us to perform an ITT analysis.

Our study has some important limitations. One was the failure to recruit sufficient caregivers. Caregivers of patients with MS experience the stress and difficulties related to the management of a relative with a chronic disabling disease. Furthermore, patients’ depression has a negative impact on the quality of life and mood of their caregivers (Giordano et al., 2012). We were therefore interested in evaluating whether caregivers would benefit from the treatment. Unfortunately, we were unable to recruit and retain sufficient caregivers to warrant statistical analysis of their data. Another limitation is that owing to the characteristics of the intervention, which requires at least partially preserved mobility, we had to exclude severely disabled patients. Similarly, the high rate of refusal to participate in the study (45% of patients that fulfilled the criteria declined to participate) and the high dropout rate may be at least partially explained by logistic factors. The CReSM Unit is the regional reference center for MS, and thus patients travel to the center from a wide area. Mobility difficulties may have prevented or discouraged MS patients living further from the center from participating in the interventions. In view of the apparent efficacy of MBIs in MS patients it is therefore essential to develop new ways of delivering mindfulness-based protocols, such as eHealth programs, to render them more accessible.

In conclusion, this study is, to the best of our knowledge, the first RCT to compare the effectiveness of a MBI and psycho-educational intervention in MS patients. Other strengths of the study in addition to the use of an active control group are the focus on symptoms of depression and the use of a clinical interview to corroborate self-report data on psychological functioning. The results on completers strengthen the evidence that MBIs reduce depressive symptoms and enhance quality of life in patients with MS (Levin et al., 2014; Simpson et al., 2014; San José et al., 2016) as they demonstrate the effects of MBIs are specific to this type of intervention and are not the result of non-specific factors. Furthermore, the BAM intervention was shown to be effective in reducing negative representations of the illness, modulating cognitive and emotional aspects of illness perception and enhancing acceptance of the pathology. The BAM intervention also led to a reduction in anxiety symptoms and perceived stress, although it was no more effective in these respects than the PEI. This finding is not surprising, given that the BAM protocol was specifically tailored to manage depression. All the improvements observed immediately after the BAM intervention were maintained at the long-term follow-up assessment thus providing further supporting for the effectiveness of the BAM intervention in MS patients.

## Ethics Statement

This study was carried out in accordance with the recommendations of the Medical Ethics Committee of San Luigi Gonzaga University Hospital with written informed consent from all subjects. All subjects gave written informed consent in accordance with the Declaration of Helsinki. The protocol was approved by the Medical Ethics Committee of San Luigi Gonzaga University Hospital.

## Author Contributions

SC, LO, and MB were responsible for the conception and design of the study. MB, DF, FS, GB, SM, and FO were responsible for data collection and for clinical evaluations and treatments. VT was responsible for data analysis. SC, RT, and LO contributed to the interpretation of data. VT and SC wrote the article, which was critically revised by all the other authors. All authors have approved the final version of the manuscript.

## Conflict of Interest Statement

The authors declare that the research was conducted in the absence of any commercial or financial relationships that could be construed as a potential conflict of interest.

## Footnotes

1 www.randomizer.org
