# Supplementary material for: The Effectiveness of a Body-Affective Mindfulness Intervention for Multiple Sclerosis Patients with Depressive Symptoms: A Randomized Controlled Clinical Trial
Source: Front Psychol. 2017 Nov 30;8:2083. doi: 10.3389/fpsyg.2017.02083 (PMC5714860; doi:10.3389/fpsyg.2017.02083)
Supplement: Supplementary file 1 [file Supplementary_Material.DOCX]

Supplementary Material

**The Effectiveness of a Body-Affective Mindfulness Intervention for Multiple Sclerosis Patients with Depressive Symptoms: a Randomized Controlled Clinical Trial**

**Sara Carletto, Valentina Tesio^*^, Martina Borghi, Diana Francone, Francesco Scavelli, Gabriella Bertino, Simona Malucchi, Antonio Bertolotto, Francesco Oliva, Riccardo Torta, Luca Ostacoli**

*** Correspondence:** Valentina Tesio,

Department of Psychology, University of Turin,

Via Po 14, 10123 Turin, Italy

E-mail: valentina.tesio@unito.it

**Table 1.** Psychopathology as assessed with the Mini International Neuropsychiatric Interview-Plus at baseline (T0), after treatment (T1) and follow-up (T2) by intervention group.

|  | **BAM (N = 36)** | | | **PEI (N = 35)** | | |
| --- | --- | --- | --- | --- | --- | --- |
|  | **T0** | **T1** | **T2** | **T0** | **T1** | **T2** |
| Adjustment disorders | 17 (47.2) | 4 (11.1) | 3 (8.3) | 10 (28.6) | 12 (34.3) | 4 (11.4) |
| Major Depressive Episode | 11 (30.6) | 3 (8.3) | 2 (5.6) | 13 (37.1) | 8 (22.9) | 4 (11.4) |
| Anxiety Disorders | 12 (33.3) | 1 (2.8) | 3 (8.3) | 7 (20.0) | 6 (17.1) | 12 (34.3) |
| Other mood disorders | 1 (2.8) | 1 (2.8) | 2 (5.6) | 2 (5.7) | 5 (14.3) | 6 (17.1) |

N (%) of patients is shown.

**Table 2.** Caregivers’ socio-demographic characteristics by intervention group.

|  | **BAM (N = 9)** | **PEI (N = 10)** |
| --- | --- | --- |
|  | **Mean (SD)** | **Mean (SD)** |
| **Age** | 61.4 (15.8) | 57.5 (15) |
| **Years of education** | 8.8 (2.6) | 11 (2.6) |
|  |  |  |
|  | **N (%)** | **N (%)** |
| **Sex (M/F)** | 0 (0) / 9 (100) | 5 (50) / 5 (50) |
| **Marital status** |  |  |
| Single | 1 (11.1) | 1 (10) |
| Married | 4 (44.4) | 7 (70) |
| Widowed | 4 (44.4) | 2 (20) |
| **Employment Status** |  |  |
| Unemployed | 6 (66.7) | 8 (80) |
| Employed | 3 (33.3) | 2 (20) |
| **Relationship to the patients** |  |  |
| Mother | 7 (77.8) | 3 (30) |
| Father |  | 1 (10) |
| Wife | 1 (11.1) | 1 (10) |
| Husband |  | 4 (40) |
| Daughter | 1 (11.1) | 1 (10) |
